# Supplementary material for: Menin‐regulated Pbk controls high fat diet‐induced compensatory beta cell proliferation
Source: EMBO Mol Med. 2021 Apr 6;13(5):e13524. doi: 10.15252/emmm.202013524 (PMC8103087; doi:10.15252/emmm.202013524)
Supplement: Supplementary file 1 — Appendix [file EMMM-13-e13524-s012.pdf]

Appendix

Content:

1. **Appendix Figure S1. Pbk gene and protein expression profiles in human and mouse.**
2. **Appendix Figure S2. PIME cells express pancreatic progenitor-related markers and with a *Men1*-exisable character**
3. **Appendix Figure S3. Body weight measurement on Pbk KI mice and control mice with MI treatment**
4. **Appendix Table S1. The information of human islet donors used in this study.**
5. **Appendix Table S2. Predicted JunD binding sites at *Pbk* locus using software PROMO3.0.**
6. **Appendix Table S3. All antibodies, reagents, commercial kits, animal and cell lines, oligoes, and software used in this study.**

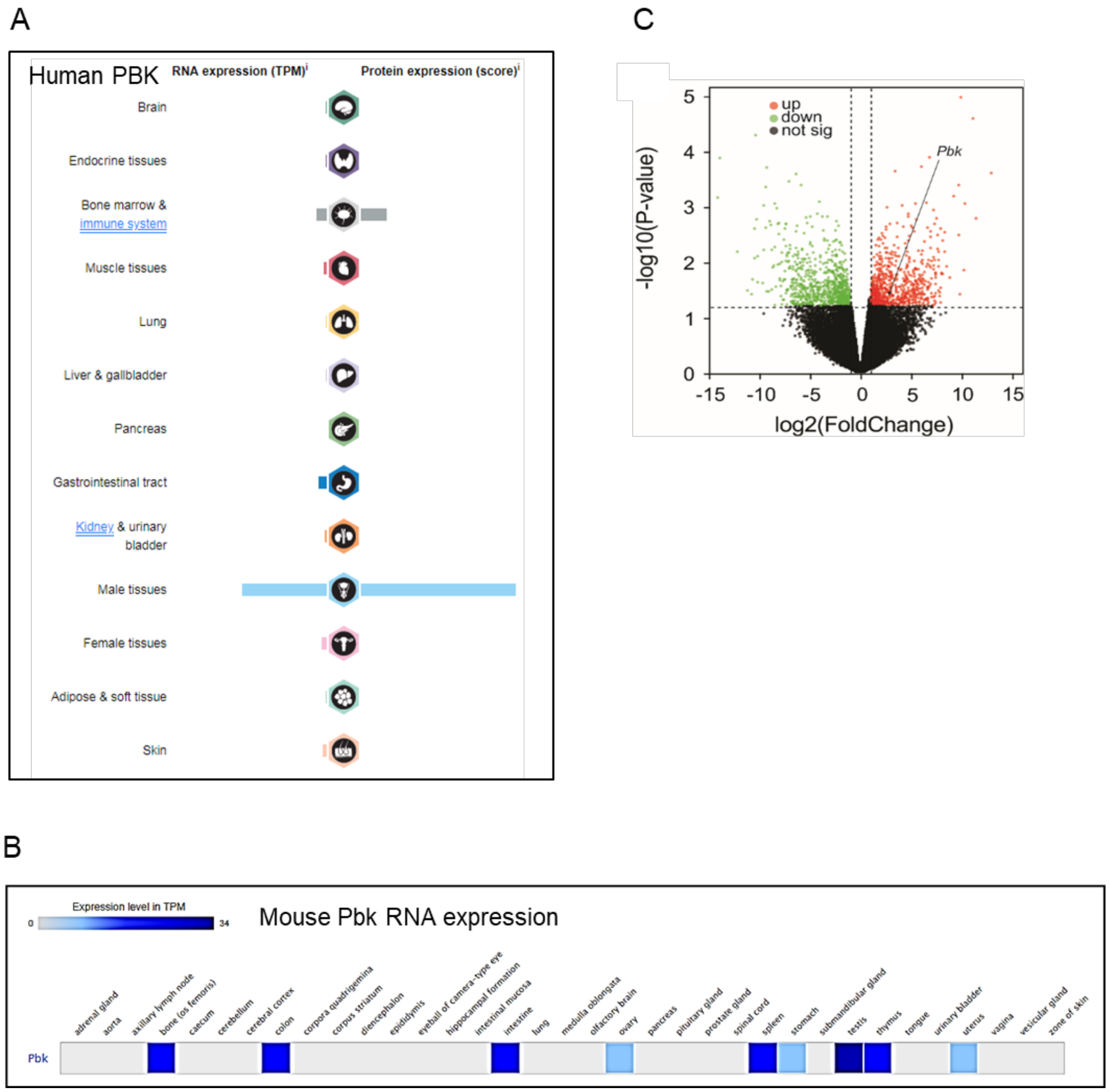

1. Appendix Figure S1. Pbk gene and protein expression profiles in human and mouse.

A. Pbk gene and protein expression profiles in human adult organs and tissues. Adapted from the Human Protein Atlas database: https://www.proteinatlas.org/ENSG00000168078-PBK/tissue.

B. Pbk gene expression profiles in different tissues of adult male mice. Adapted from Expression Atlas database: https://www.ebi.ac.uk/gxa/experiments/E-MTAB-3579/Results?geneQuery=%5B%7B"value"%3A"ensmusg00000022033"%7D%5D&filterFactors=%7B"DEVELOPMENTAL_STAGE"%3A%5B"adult"%5D%7D.

C. Volcano plot showing the fold change (y-axis) versus adjusted (adj.) p-value (x-axis) of the pancreatic islet transcriptomes between chow and HFD-fed rats (30 days). Pbk gene expression level is indicated by the arrow. Genes highlighted in red or green are based on the thresholds of Log2 fold change > 1 and adj. p value < 0.01. GSE number of this cDNA Microarray library is GSE44047.

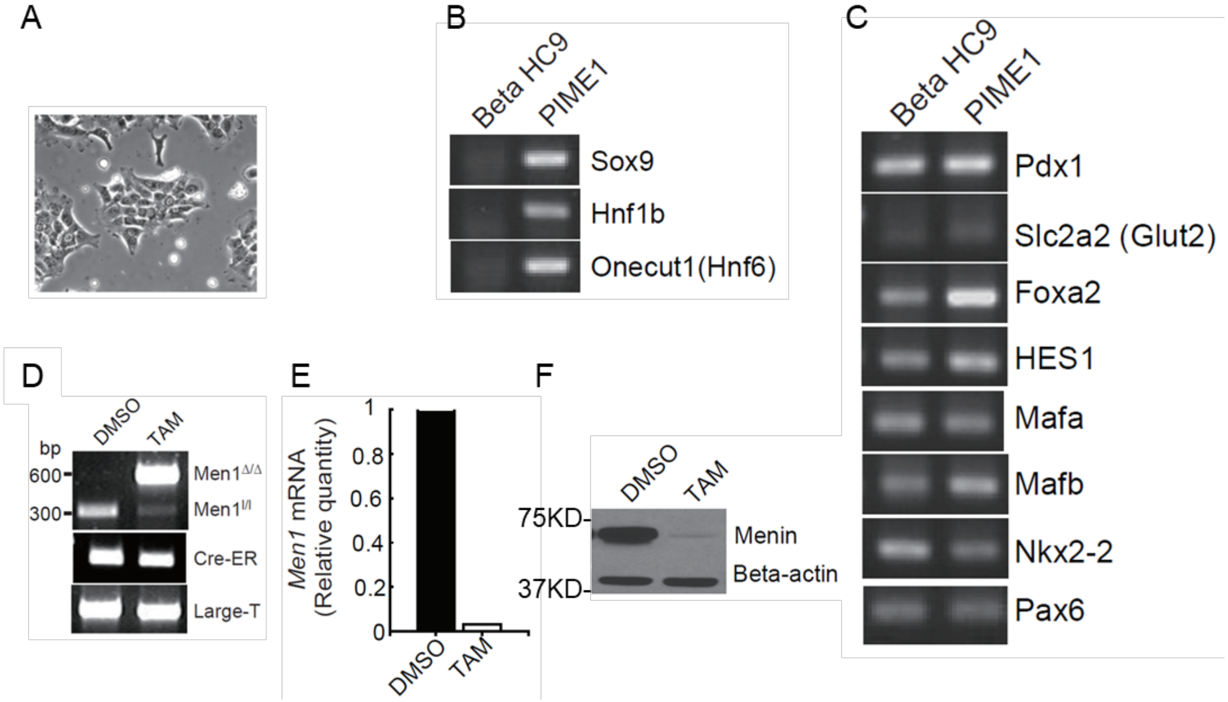

2. Appendix Figure S2. PIME cells express pancreatic progenitor-related markers and with a *Men1*-exisable character

A. The morphological observation of PIME cells. Magnification folds is 4×20.

B. PIME cells showed higher expression profile of pancreatic progenitor related genes, such as Sox9, Hnf1b, and Onecut1(Hnf6) than a beta cell line, beta HC9.

C. PIME cells and Beta HC9 cells owned similar beta cell related gene expression profiles.

D. Tamoxifen (TAM) treatment-induced *Men1* gene excision in PIME cells was verified by PCR.

E, F. TAM treatment-induced downregulation of *Men1* gene expression in PIME cells was verified by qPCR (E) and WB (F).

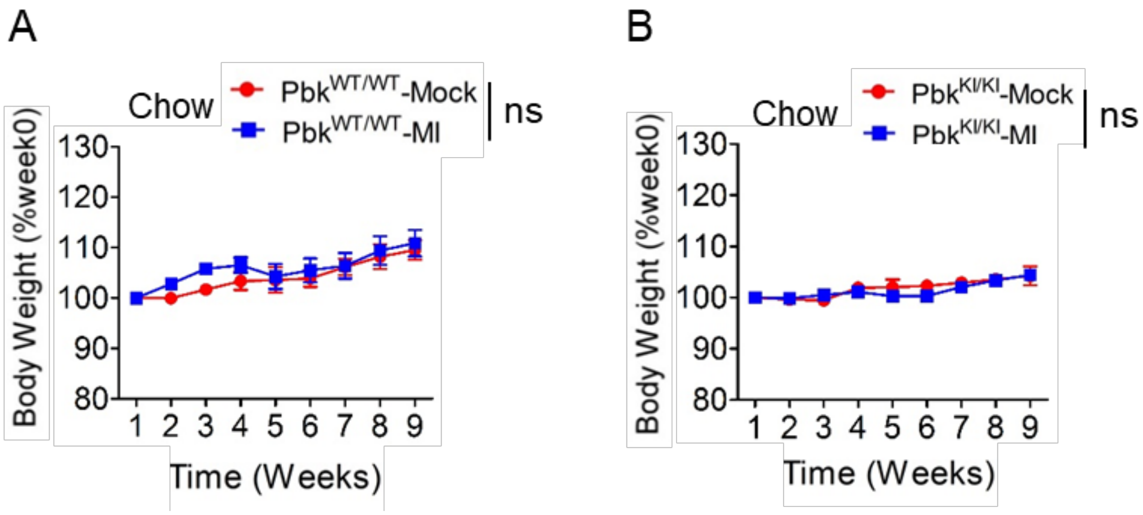

3. Appendix Figure S3. Body weight measurement on Pbk KI mice and control mice with MI treatment

A, B. Body weight measurement of $Pbk^{WT/WT}$ (A) and $Pbk^{KI/KI}$ (B) mice treated with MI on chow diet. ns, not statistically significant difference (Two-way ANOVA). $P = 0.2925$ (A), $P = 0.5739$ (B).

4. Appendix Table S1. The information of human islet donors used in this study.

ID	Age	Gender	Height	BW （kg）	Race	BMI	HbA1c (%)	Cause of death
#1	40	Female	67”	91	Hispanic	31.8	4.8	Stroke
#2	43	Female	64”	82	Caucasian	31.5	5.0	Anoxic event
#3	56	Male	68’’	101	Caucasian	34.0	5.5	Gunshot wound
#4	54	Male	71”	72	Caucasian	22.3	5.8	Stroke
#5	35	Male	63”	63	Hispanic	24	4.8	Anoxic event
#6	45	Male	67”	76	Hispanic	25.0	5.1	Anoxic event

5. Appendix Table S2. Predicted JunD binding sites at *Pbk* locus using software PROMO3.0.

Predicted binding sites (Dissimilarity<5%)	Sequence	Position (-2000-+500)
Site1	GATGTCA	-1865 - -1859
Site2	TGGGTCA	-1737- -1731
Site3	GAGGTCA	-791- -785
Site4	TGACACC	-579- -573
Site5	GGGGTCA	340- 346

6. **Appendix Table S3.** All antibodies, reagents, commercial kits, animal and cell lines, oligoes, and software used in this study.

**Reagent or Resource**	**Source**	**Identifier**
**Antibodies**		
Mouse monoclonal anti-Insulin	Cell Signaling Technology	Cat# 8138S
Mouse monoclonal anti-PBK	BD	Cat# 612170
Rabbit polyclonal anti-PBK	Abcam	Cat# ab226923
Rat monoclonal anti-BrdU	Abcam	Cat# ab6326
Rabbit polyclonal anti-Menin-CHIP	Bethyl Laboratories	Cat# A300-105A
Rabbit monoclonal anti-P38	Cell Signaling Technology	Cat# 8690S
Rabbit polyclonal anti-P-P38	Cell Signaling Technology	Cat# #9211S
Rabbit monoclonal anti-P-SAPK/JNK	Cell Signaling Technology	Cat# 4668T
Rabbit polyclonal anti- SAPK/JNK	Cell Signaling Technology	Cat# 9252T
Rabbit monoclonal anti-ERK1/2	Cell Signaling Technology	Cat# 4695S
Rabbit monoclonal anti-P-ERK1/2	Cell Signaling Technology	Cat# 4370S
Rabbit polyclonal anti-P-JunD	Cell Signaling Technology	Cat# 9164S
Rabbit polyclonal anti-CcnB1	Cell Signaling Technology	Cat# 4138T
Rabbit polyclonal anti-HDAC1-GHIP	Abcam	Cat# ab7028
Rabbit polyclonal anti-HDAC3-GHIP	Abcam	Cat# ab7030
Rabbit polyclonal anti-JunD-CHIP	Santa Cruz	Cat# Sc-74
Rabbit polyclonal anti-Histone H3 (acetyl K9)-CHIP	Abcam	Cat# ab4441
Rabbit polyclonal anti-Mouse IgG H&L	Abcam	Cat# ab46540
Rabbit polyclonal anti-Ki67	Abcam	Cat# ab15580
Mouse monoclonal anti-bate actin	Sigma	Cat# A5441
Goat Anti-Rabbit IgG (H + L)-HRP	BIO-Rad	Cat# 1706515
Goat Anti-Mouse IgG (H+L)-HRP	BIO-Rad	Cat# 1721011
Donkey anti-Rabbit IgG Cy5	Jackson ImmunoResearch	Code:711-175-152
Donkey anti-mouse IgG Cy3	Jackson ImmunoResearch	Code:715-165-151
Alexa Fluor 546 goat anti-rabbit IgG(H+L)	Life technologies	Cat# A11035
Goat anti-Rabbit IgG-488	ThermoFisher	Cat# A-11008
Goat anti-mouse IgG-488	ThermoFisher	Cat# A-11001
**Biological Samples**		

$Pbk^{WT/WT}$ mouse islets and FFPE pancreas sections	This paper	N/A
$Pbk^{KI/KI}$ mouse islets and FFPE pancreas sections	This paper	N/A
DIO mice pancreas section	This paper	N/A
Control mice pancreas section	This paper	N/A
Human donor islets, see S Table 2	Prodo Labs	Cat# HP-19205-01
**Chemicals and other reagents**		
DPBS(10×)	Gibco	Cat# 14200-075
Non-fat dry Milk	Lab scientific	Cat# M0842
RPMI Medium 1640	Gibco	Cat# 11875-085
DMEM	Gibco	Cat# 11965-084
Prodo islet medium （Recover）	Prodo Labs	Cat# PIM-R001GMP
Pen Strep	Gibco	Cat# 15140-122
2-Mercaptoethanol(1000 X)	Gibco	Cat# 21985-023
Sodiun Pyruvate (100mM)	Gibco	Cat# 11360-070
HEPES (1M)	Gibco	Cat# 15630-080
L-Glutamine (200mM)	Gibco	Cat# 25030-081
DMSO	Sigma	Cat# D2650-100ML
Fetal Bovine Serum	Sigma	Cat# F0926-500mL
4-12% Bis-Tris Plus Blot SDS-PAGE gels	GenScript	Cat# M42012L
20×MES Blot Running buffer	GenScript	Cat# c31381608
PVDF membrane	Invitrogen	Cat# LC2002
Ultra-Pure Agarose	Invitrogen	Cat# 16500100
Precision plus protein dual color standers	BioRad	Cat# 1610374
RIPA buffer	Sigma	Cat# R0278-500ML
NuPAGE@LDS sample buffer(4X)	NOVES	Cat# 1658669
2-Mercaptoethanol	Fisher Scientific	Cat# O3446I-100
NP-40 (0.8%)	USB	Cat# 19628
Tris–HCl (pH 7.4)	Fisher BioReagents	Cat# CAS-1185-53-1
NaCl (150 mM)	Sigma	Cat# S6191
Glycerol (10%)	Sigma	Cat# G9012
Tween 20	Bio-Rad	Cat# 1706531
Triton X-100	Sigma	Cat# 9002-93-1
D-Glucose	Sigma	Cat# G6152
Human insulin	Novolin-R	Cat# 183302
PBK protein	MyBioSource	Cat# MBS145337
ATP	Sigma	Cat#11140965001
TAM	Sigma	Cat# 579002
DAPI	Thermo Fisher Scientific	Cat# 62248
Propidium iodide	Life technologies	Cat# P3566
Antigen unmasking solution	Vector Laboratories	Cat# H-3301
Coin oil	Sigma	Cat# M8823-1ML
RNase A	Sigma	Cat# R6148-25ML
Paraformaldehyde Solution, 4% in	Affymetrix	Cat# 19943

PBS		
Xylene	Polysciences	Cat# 24770-1
Ethanol	Decon Labs	Cat# 64-17-5
Methanol	Fisher Scientific	Cat# A412-4
Chloroform	Fisher Scientific	Cat# C606-1
**Critical Commercial Assays**		
Mouse insulin Elisa kit	Crystal Chem	Cat# 90080
QuantiTect SYBR Green PCR Kit	Qiagen	Cat# 204143
SuperScript® III Reverse Transcriptase	Invitrogen	Cat# 18080044
RNeasy Mini Kit (250)	Qiagen	Cat# 75142
Dual-Luciferase® Reporter Assay System	Promega	Cat# E1910
Site-Directed Mutagenesis Kit	Agilent Technologies	Cat# 200521-5
Anti-Flag Magnetic Beads	Sigma-Aldrich	Cat# M8823-1ML
MTS assay		
REDEXTRACT-N-AMP PCR REACTION MIX	Sigma	Cat# R4775-1.2ML
Pierce™ BCA Protein Assay Kit	Thermo Scientific	Cat# 23225
ECL Western Blotting Detection Reagents	Amershan	Cat# RON2106
**Experimental Models: Cell lines**		
PIME	This paper	NA
INS-1	Gift from Dr. Ma	(Kong X., et al., *Endocrinology*, 2014, 155 4676-4685)
HEK293T	ATCC	Cat# CRL-11268
**Experimental Models: Organisms/Strains**		
C57BL6/J	The Jackson Laboratory	Cat# 00664
DIO	The Jackson Laboratory	Cat# 380050
DIO-control mice	The Jackson Laboratory	Cat# 380056
$Pbk^{KI/KI}$	This paper	NA
**Inhibitor (compounds)**		
MI-503	Wuxi Pharmacy	ID:MI-503-001
MI-463	Wuxi Pharmacy	ID:MI-463-002
OTS-514	Selleckchem	Cat# S7652
T5224	APExBIO	Cat# B4664
Proteinase inhibitor cocktail	Sigma	Cat# P8340-1ML
Phostop Phosphotase Inhibitor	Roche	Cat# 4906837001
**Oligonucleotides**		
Mouse Pbk-target sgRNA	This paper	TAAAAGACTTATCTTTTTCAGTTTTAGAGCTAGAAATAGCAAGTTAAAATAAGGCTAGTCCGTTATCAACTTGAAAAAGTGGCACCGAGTCGGTGCTTTTTT
Repaired DNA for Pbk KI mouse	This paper	CTTAGCTTCATCAGTTAGTCTCTTCTGATACACAGTTCGATAATGATCATCGCATAAAAGACTTATGGCAGCGACAGCCCAAGGAGAATGAGACAACCCTCTTGGAGATCTAAAATATACATATGTAA

mouse/Rat Pbk-F	This paper	TTGCTATGGAGTATGGAGGTG
mouse/Rat Pbk-R	This paper	GATACTTTAGCCCTCTTGCCA
Mouse actin-F		CTGTCCCTGTATGCCTCTG
Mouse actin-R		ATGTCACGCACGATTTCC
human Pbk gene-F	This paper	GAAGAGGACTGAGAGTGGCT
human Pbk gene-R	This paper	CTTCTGCATAAACGGAGAGGC
human Ki67 gene-F	This paper	TACGTGAACAGGAGCCAGCA
human Ki67 gene-R	This paper	GTTCCCTGAGCAACACTGTC
human P27 gene-F	This paper	GGAGCAATGCGCAGGAATAA
human P27 gene-R	This paper	TGGGGAACCGTCTGAAACAT
human P18 gene-F	This paper	ATGGATTTGGAAGGACTGCG
human P18 gene-R	This paper	ATGACAGCGAAACCAGTTCG
Human actin-F		GGTCATCACCATTGGCAATGA
Human actin-R		GCACTGTGTTGGCGTACA
Mouse Pbk-CHIP-primer-F	This paper	CAGAGCCTGGCCTTCTGATTT
Mouse Pbk-CHIP-primer-R	This paper	GTGTGTGTGTGTGTTTCTGCT
Rat Pbk-CHIP-primer-F	This paper	CCAGCCCCGAATAATGAAACT
Rat Pbk-CHIP-primer-R	This paper	CCCTTACCTAGGAAGCGCAAG
**Recombinant DNA**		
pLX304-human Pbk	This paper	N/A
pLPCX-human JunD-Flag	This paper	N/A
pLG3.0-JBS-4-WT-luc	This paper	GAGCTCTGACACCTGACACCTGACACCAGATC T
pLG3.0-JBS-4-MUT-luc	This paper	GAGCTCAAAAAAAAAAAAAAAAAAAAAAGATCT
shRNA targeting sequence: Rat Pbk #1	This paper	GCTATGGAGTATGGAGGTGAA
shRNA targeting sequence: Rat Pbk #5	This paper	CCTTACTCTGTGGGAAATGAT
shRNA targeting sequence: Mousr/Rat JunD #1	This paper	GAGAAAGTCAAGACCCTCAAA
shRNA targeting sequence: Mouse JunD #2	This paper	CGCCGGATCTTGGGCTGCTCA
shRNA targeting sequence: Rat JunD #2	This paper	GCGCCTGGAGGAGAAAGTCAA
sgRNA targeting sequence: Rat menin #1	This paper	CATGCGCTGTGACCGCAAGA
Erk2 Bacterial Expression plasmid	Addgene	Cat # 29582
**Software**		
FACSDiva V3.0	BD Biosciences	NA
FlowJo 10.3	FlowJo	N/A
GrashPad Prism version 4.0	GraphPad Software	N/A
R (3.5.0)		http://www.R-project.org
Image J-win64	ImageJ	https://imagej.net/Fiji/Downloads
