# Supplementary material for: Menin‐regulated Pbk controls high fat diet‐induced compensatory beta cell proliferation
Source: EMBO Mol Med. 2021 Apr 6;13(5):e13524. doi: 10.15252/emmm.202013524 (PMC8103087; doi:10.15252/emmm.202013524)
Supplement: Supplementary file 2 — Expanded View Figures PDF [file EMMM-13-e13524-s004.pdf]

Expanded View Figures

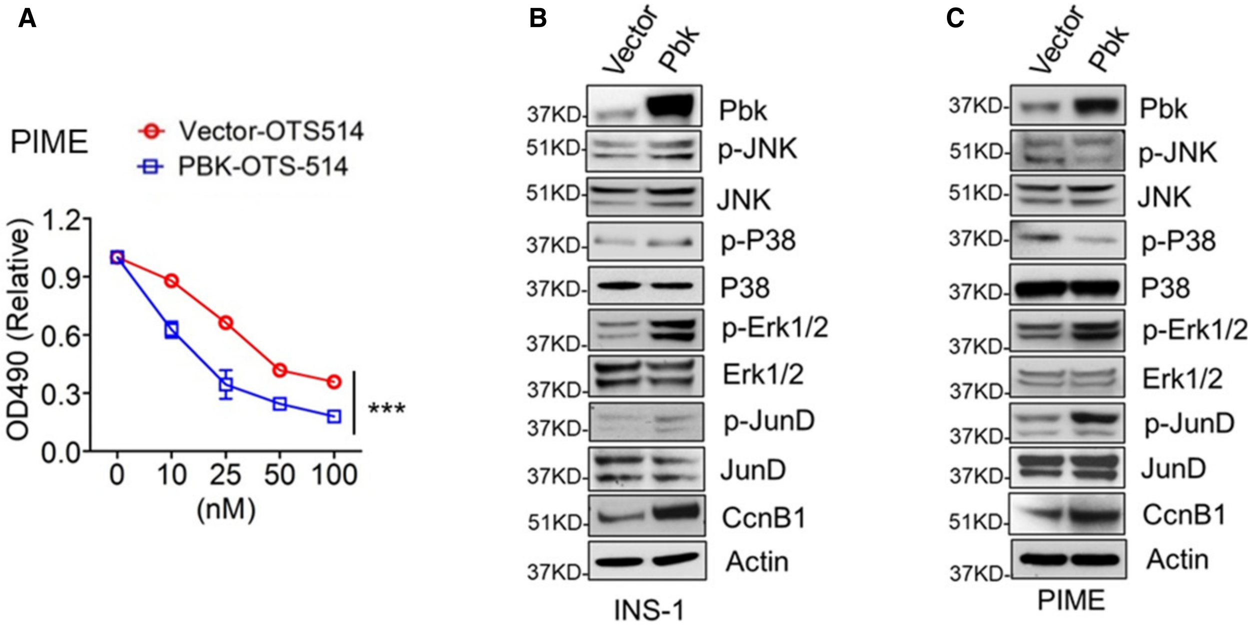

Figure EV1. Ectopic expression of Pbk activates cell growth-related intracellular signaling pathways.

A Pbk overexpression PIME cells and control cells were treated by OTS 514 with different concentrations for 72 h. The cell growth was assessed by MTS assay. Five independent replicates (n = 5). Data are represented as mean ± SEM. ***P = 0.0004 (Two-way ANOVA).

B, C The effect of Pbk overexpression on phosphorylation levels of JNK, P38, ERK1/2, and JunD, as well as total protein level of CcnB1 in INS-1 cells (B) and PIME cells (C).

Figure EV2. Body weight, insulin tolerance, and glucose tolerance in Pbk kinase mutant KI mice.

A, B Body weight measurement of male (A) or female (B) $Pbk^{KI/KI}$ and $Pbk^{WT/WT}$ mice (n = 5 for male mice group and n = 3 for female mice group) from 2 to 13 weeks after birth. ns, not statistically significant difference (Two-way ANOVA).

C Body weight measurement of $Pbk^{WT/WT}$ and $Pbk^{KI/KI}$ mice on HFD (n = 3 for each group). ns, not statistically significant difference (Two-way ANOVA).

D ITT was performed on $Pbk^{KI/KI}$ and $Pbk^{WT/WT}$ mice during the 8th week of HFD feeding (n = 3 for each group). ns, not statistically significant difference (Two-way ANOVA).

E–H Persistent measurement of GTT for $Pbk^{KI/KI}$ or $Pbk^{WT/WT}$ mice at various times, i.e., 0, 3rd, 5th, 8th, and 12th week during HFD feeding (n = 4 for each group). Dot line, fasting blood glucose number > 150 was designated as hyperglycemia. **P = 0.0022, ***P < 0.0001 (Two-way ANOVA), ns, not statistically significant difference.

Data information: Data are represented as mean ± SEM.

© 2021 The Authors

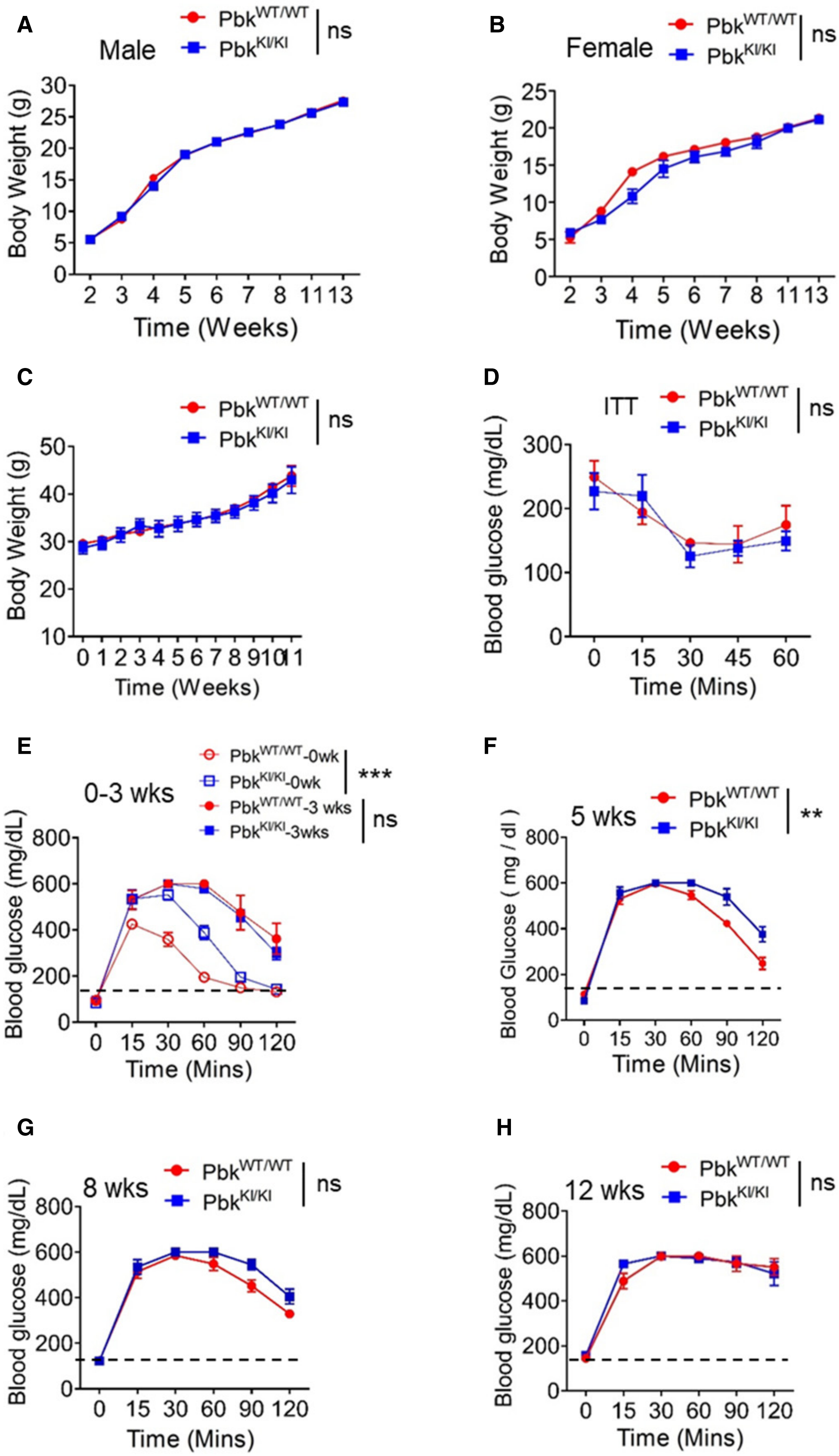

Figure EV2.

 © 2021 The Authors

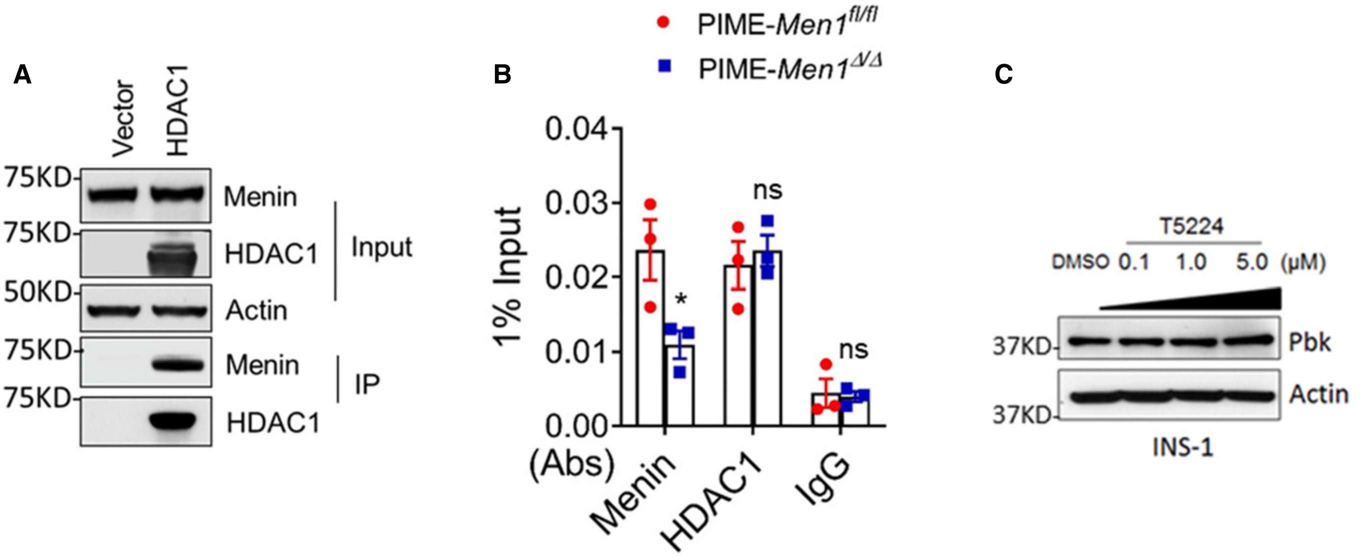

Figure EV3. HDAC1 binds to menin but does not participates in menin-mediated Pbk expression suppression.

A Co-IP experiments in HEK293T cells transfected with vector and flag-HDAC1 expression plasmids. WB data showed the level of menin and HDAC1 of input samples and output samples.

B PIME and PIME menin-null cells were subjected to ChIP assay to detect for the binding of menin and HDAC1 at the *Pbk* locus. Three independent experiments (n = 3). *P = 0.0469 (two-tailed unpaired Student's t-test), ns, not statistically significant difference.

C INS-1cells were treated with various doses of T5224 for 48 h, followed by detecting Pbk expression using WB.

Data information: Data are represented as mean ± SEM.

© 2021 The Authors

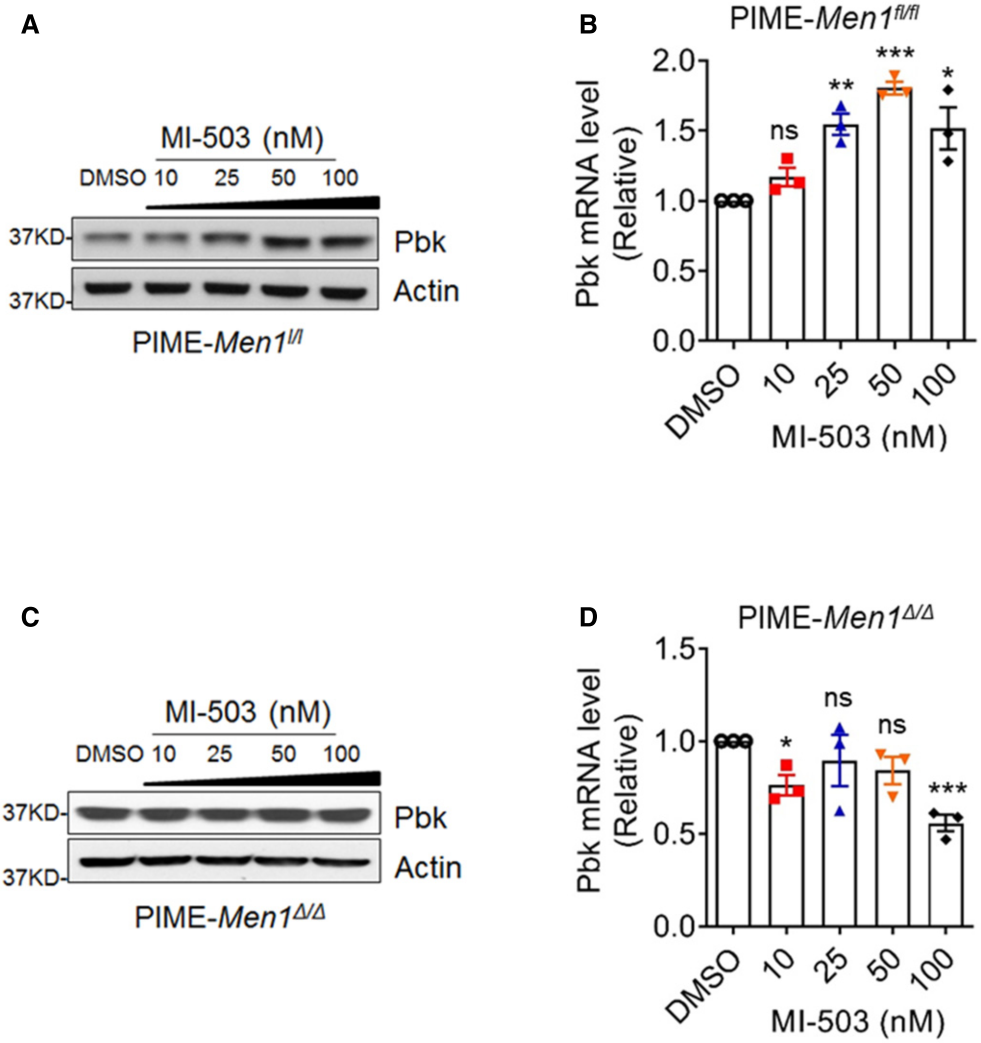

Figure EV4. MI-503 promotes Pbk expression in PIME cells.

A, B PIME cells were treated with various doses of MI-503 for 48 h, followed by detecting Pbk expression using WB (A) and qPCR (B). qPCR data were from three independent experiments (n = 3). **P = 0.0019, ***P < 0.0001, *P = 0.0253 (two-tailed unpaired Student's t-test), ns, not statistically significant difference.

C, D Menin-null PIME cells were treated with various concentrations of MI-503 for 48 h, followed by detecting Pbk expression with WB (C) and qPCR (D). qPCR data were from three independent experiments (n = 3). *P = 0.0126, ***P = 0.0006, (two-tailed unpaired Student's t-test), ns, not statistically significant difference.

Data information: Data are represented as mean ± SEM.

 © 2021 The Authors

Figure EV5. The physiological parameters of HFD-induced diabetic mice treated with MI.

A, B IPGTTs (glucose at 2 g/kg of body weight) were performed during 12-week MI administration at different times indicated. Blood glucose levels were measured and showed for vehicle (A)- or MI-403 (B)-treated mice, $n = 5$ for each group of mice. Dot line, fasting blood glucose number > 150 was designated as hyperglycemia.

C–G IPGTTs (glucose at 2 g/kg of body weight) were performed during 12-week MI administration at different times indicated. Blood glucose levels were measured and showed. Solid red triangle, vehicle-treated control mice ($n = 5$); Solid blue circle, MI-463-treated DIO mice ($n = 5$). Dot line, fasting blood glucose number > 150 was designated as hyperglycemia. $^{**}P = 0.0027$ (E), $^{***}P < 0.0001$ (F), $^{**}P = 0.0031$ (G) (Two-way ANOVA), ns, not statistically significant difference.

H Body weight of DIO mice with MI-463 ($n = 5$) or vehicle ($n = 5$) administration at 70 mg/kg by gavage daily for 12 weeks. ns, not statistically significant difference (Two-way ANOVA).

I ITT was performed in MI-463 ($n = 5$)- or vehicle ($n = 5$)-treated mice before and at the end of MI-463 treatment. $^{**}P = 0.0029$ (Two-way ANOVA).

J Average daily food intake for MI-463 ($n = 5$) or vehicle ($n = 5$)-administered mice was calculated from weekly food consumption (for each cage) over average body weight of all mice in a cage in first three weeks with MI administration. ns, not statistically significant difference (two-tailed unpaired Student's *t*-test).

K Adiposity index in the vehicle or MI-463-treated DIO mice for 12 weeks ($n = 5$ for each group). ns, not statistically significant difference (two-tailed unpaired Student's *t*-test).

L, M Body weight measurement of $Pbk^{WT/WT}$ (L) and $Pbk^{KI/KI}$ (M) mice ($n = 5$ for each group) administrated with vehicle or MI on HFD. ns, not statistically significant difference (Two-way ANOVA).

Data information: Data are represented as mean ± SEM.

© 2021 The Authors

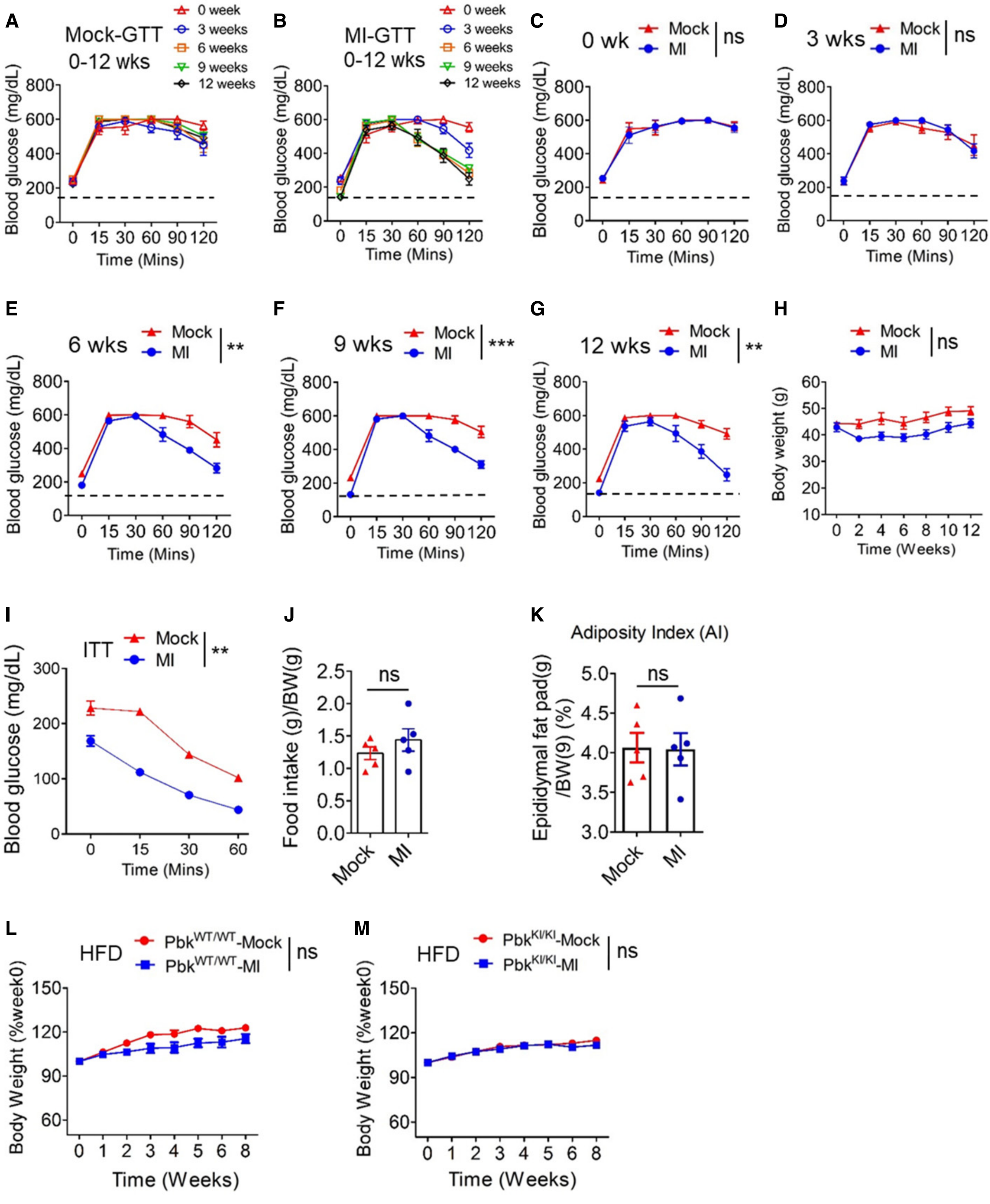
A
Mock-GTT
0-12 wks
0 week
3 weeks
6 weeks
9 weeks
12 weeks
Blood glucose (mg/dL)
Time (Mins)
B
MI-GTT
0-12 wks
C
0 wk
Mock
MI
ns
D
3 wks
E
6 wks
**
F
9 wks
***
G
12 wks
H
Body weight (g)
Time (Weeks)
I
ITT
J
Food intake (g)/BW(g)
K
Adiposity Index (AI)
Epididymal fat pad(g) /BW(9) (%)
L
HFD
Pbk^WT/WT-Mock
Pbk^WT/WT-MI
Body Weight (%week0)
M
Pbk^KI/KI-Mock
Pbk^KI/KI-MI

Figure EV5.
